# Sudden Tracheal Collapse during EGD and Subsequent Anesthetic Management with Dexmedetomidine-Ketamine in a Patient with Achalasia and Tracheomalacia

**DOI:** 10.1155/2011/281679

**Published:** 2011-11-09

**Authors:** Joshua H. Atkins, Jeff E. Mandel, David C. Metz

**Affiliations:** ^1^Department of Anesthesiology and Critical Care and Department of Otorhinolaryngology, Perelman School of Medicine at the University of Pennsylvania, 680 Dulles Bldg, 3400 Spruce Street, Philadelphia, PA 19104, USA; ^2^Department of Anesthesiology and Critical Care, Perelman School of Medicine at the University of Pennsylvania, 680 Dulles Bldg, 3400 Spruce Street, Philadelphia, PA 19104, USA; ^3^Division of Gastroenterology, Department of Medicine, Perelman Center for Advanced Medicine, Perelman School of Medicine at the University of Pennsylvania, Fourth Level, Suite 4-370S, 400 Civic Center Boulevard, Philadelphia, PA 19104, USA

## Abstract

We present a patient who experienced airway obstruction during an elective esophagogastroduodenoscopy (EGD) under anesthesia secondary to previously undiagnosed tracheomalacia. Physiology of airway obstruction with forced breathing maneuvers is discussed along with the potential advantages of dexmedetomidine-ketamine sedation for management of patients with achalasia undergoing outpatient endoscopic procedures.

## 1. Introduction

Patients with achalasia routinely present to the outpatient endoscopy suite for EGD with or without therapeutic BOTOX injection under sedation. Achalasia may be rarely associated with airway obstruction from megaesophagus. Sudden obstruction secondary to tracheomalacia with dynamic airway collapse has not been reported previously. Diverse options exist for the anesthetic management of such cases, and the utility of dexmedetomidine for sedation in medically complex patients is increasingly evident.

## 2. Case Report

**Figure 1:**
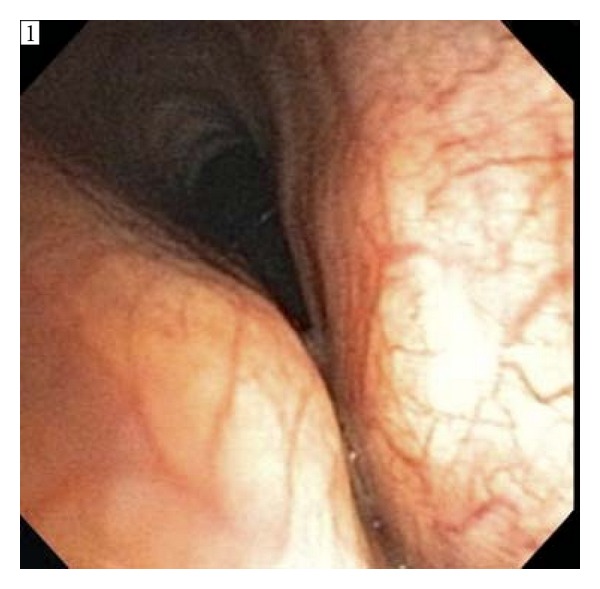
Trachea during bronchoscopic examination demonstrating tracheomalacia.

An 85-year-old gentleman (73 kg, 180.3 cm) with severe, symptomatic achalasia felt to be a poor surgical risk and therefore a poor candidate for surgical myotomy or pneumatic dilatation presented to the outpatient ambulatory endoscopy center for EGD with esophageal BOTOX injection under anesthesia after previously undergoing an uncomplicated similar procedure some months before. Prior to the procedure, he was kept nil per os (NPO) for 18 hours to promote esophageal drainage. Standard monitors (SpO_2_, ECG, and noninvasive BP) and nasal cannula oxygen (4 L) with capnometry were applied. Intravenous midazolam (1 mg) and glycopyrrolate (0.2 mg) were administered, the patient was placed in the left-lateral decubitus position, and an oral bite block was inserted. A bolus of 6 mL of ketofol (propofol 10 mg/mL with ketamine 1 mg/mL) was given and the procedure started. Spontaneous ventilation, as assessed by observation and capnometry, was maintained. The endoscope passed smoothly into the proximal esophagus without a patient response, but upon esophageal insufflation a bolus of solid food matter was propelled into the oropharynx accompanied by active patient coughing. Prompt and thorough oropharyngeal suctioning was performed along with a jaw thrust and chin lift. The patient continued to make respiratory efforts, but no air exchange was detected by capnometry, and paradoxical chest wall movement was observed. Passage of a lubricated nasal trumpet and placement of a Guedel airway did not alleviate the obstruction, and significant oxygen desaturation occurred. The procedure was abandoned. Face mask oxygen and gentle positive pressure via bag-mask ventilation promptly restored gas exchange and oxygen saturation to >95%, and the patient began to emerge from anesthesia. Brief flexible fiberoptic examination of the glottic opening via the right naris revealed no particulate matter or other obstruction in the glottic inlet. Under light sedation and topicalization of the oropharynx, a pulmonologist performed a complete flexible bronchoscopic exam that revealed extensive tracheomalacia from the vocal cords to the carina, including portions of the right mainstem bronchus (Figure 1). Dynamic tracheal collapse was evident during spontaneous ventilation involving the anterolateral but not posterior wall. The patient emerged from anesthesia and was transported to the recovery room on nasal cannula oxygen. He was awake and alert with stable vital signs and SpO_2_ 94–99%. Chest CT performed shortly after the aborted procedure revealed mild inflammation and an enlarged sigmoid-shaped esophagus consistent with long-standing achalasia. Dynamic airway maneuvers to assess tracheal diameters were not performed.

The patient was rescheduled for EGD and BOTOX injection after 48 hours of NPO. He was brought to the procedure room at which time standard monitors and nasal cannula oxygen with capnometry were applied. The patient was placed in the left recumbent position and glycopyrrolate 0.2 mg IV was administered followed by dexmedetomidine 1.5 mcg/kg over fifteen minutes then infusion at 0.7 mcg/kg/h. Topical anesthesia of the right naris was accomplished with lidocaine. An intravenous bolus of 0.5 mg/kg ketamine was given followed by infusion at 1 mcg/kg/min. A flexible pediatric fiberoptic bronchoscope was passed via the right naris to obtain a clear view of the vocal cords, and smooth passage of the endoscope into the esophagus was also visualized. The patient demonstrated minimal response to either procedure, while spontaneous ventilation and oxygenation were maintained. The residual liquid contents of the dilated esophagus were briskly evacuated, and 100 units of BOTOX were injected into the gastroesophageal junction in divided doses. There was no evidence of proximal airway obstruction from the limited view via the fiberscope. The patient coughed several times in response to secretions reaching the arytenoid cartilages suggesting preservation of protective airway reflexes under the sedation regimen. The procedure was completed smoothly, and the patient was awake and conversant within ten minutes of the cessation of infusions. During the procedure, there was no evidence of dynamic airway collapse: continuous capnographic evidence of CO_2_ was present and the patient demonstrated no hemodynamic instability, respiratory depression, or decrease in oxygen saturation. The patient was discharged to outpatient followup with a plan for repeat EGD in due course and evaluation for possible nighttime CPAP delivery.

## 3. Discussion

To our knowledge, this is the first clinical report of dynamic airway collapse secondary to undiagnosed tracheomalacia in a patient with advanced achalasia.

Achalasia is an uncommon motility disorder of the esophagus that develops secondary to degeneration of inhibitory neurons in the enteric plexi. The clinical result is aperistalsis of the lower esophageal body and incomplete relaxation of the lower esophageal sphincter due to unbalanced activity of excitatory neurons. The resulting slow passage of solid and liquid food presents clinically as dysphagia with retained food matter and megaesophagus [1].

Treatment with BOTOX injection to relax the lower esophageal sphincter has become the mainstay of achalasia therapy in patients deemed to be unfit for surgical myotomy or pneumatic dilation and is often also employed early in the disease process as a diagnostic trial. Endoscopic BOTOX injection is commonly performed under nurse-administered midazolam/fentanyl sedation in the outpatient GI suite though airway protection is often a concern, especially in patients with massively dilated esophagi as in the case under discussion. For patients who fail traditional sedation, a variety of approaches are employed ranging from propofol sedation to endotracheal intubation with rapid sequence induction. Indeed, prior to these encounters, our patient was variably managed with nurse-administered sedation, endotracheal intubation, and propofol-only deep sedation for similar procedures. Although uncommon, airway obstruction secondary to massive esophageal dilation with resulting posterior tracheal wall compression has been reported in children and adults with achalasia [2, 3]. Management requires immediate tracheal intubation, positive pressure ventilation, and esophageal decompression.

Acquired adult tracheobronchomalacia has many etiologies but is commonly idiopathic or secondary to chronic inflammation from recurrent infection and often presents in later life [4]. Symptoms may not be present until late in the disease process when degeneration of the tracheal cartilages is advanced. Presentation can often be confused with symptomatic asthma, and the presentation is further muddled by concomitant achalasia. Airway collapse results in changes in transtracheal pressure gradients that cannot be compensated for by the weakened tracheal wall. Unmasking of tracheomalacia under general anesthesia has been described previously [5].

Without prior knowledge of tracheomalacia in this patient, propofol-ketamine was selected for the sedation. The addition of ketamine may reduce the total dose of propofol required while also providing analgesic properties that blunt the response to endoscopy. When used as a sole agent, ketamine is believed to preserve airway reflexes, but the ability of low-dose ketamine to potentiate the depressive effects of propofol on these reflexes has not been formally studied. In our typical patient, a small loading dose of the ketofol mixture (0.5–0.8 mg/kg) is administered followed by expedient passage of the endoscope into the esophagus with prompt aspiration of any residual contents by the endoscopist.

In this patient, passive regurgitation triggered a robust and protective cough reflex that persisted and was accompanied by airway obstruction not relieved by standard interventions. Reopening of the airway required positive pressure mask ventilation, which was consistent with dynamic upper or lower airway collapse. 

We considered three primary mechanisms for the observed phenomenon. These are (1) posterior tracheal collapse from external esophageal compression; (2) upper airway with concomitant extrathoracic tracheal obstruction secondary to the Müeller maneuver; (3) intrathoracic tracheal collapse due to repeated Valsalva maneuver. Laryngospasm from insufficient depth of anesthesia was a consideration; however, the ability to mask ventilate immediately with positive pressure and the observed tracheomalacia during bronchoscopy make this possibility significantly less compelling. The first mechanism has been reported on several occasions in the literature [6]. However, the bronchoscopic finding of diffuse tracheobronchomalacia of the anterolateral wall decreases the likelihood of esophagus-mediated compression.

The Müeller maneuver is used clinically in the diagnosis of obstructive sleep apnea in the awake patient, as described in early literature by Sher and colleagues [7]. The maneuver is characterized by inspiration from functional residual capacity (FRC) against a closed airway (i.e., closed mouth and nares) that results in airway collapse at the hypopharynx, base of tongue, or lateral pharyngeal wall as detected by manometry or fiberoptic nasal endoscopy [8]. We hypothesize that only extrathoracic areas of weakened trachea or pharyngeal soft tissue would demonstrate collapse with the Mueller maneuver. Moreover, relief of obstruction was attempted with multiple interventions (nasal trumpet, jaw thrust, and Guedel airway) that should have facilitated the opening of the proximal airways.

A forced expiratory maneuver is a part of the CT airway diagnostics for tracheomalacia [9]. In contrast, the Valsalva maneuver is a forced expiration against a closed glottis. A Valsalva maneuver significantly increases intrathoracic pressure against a closed glottis such that areas of intrathoracic tracheomalacia will collapse. Cough, defined as forced expiration against a closed glottis after inspiration, would result in a similar clinical picture. Suto and Tanabe employed MRI to demonstrate that coughing decreased tracheal cross-sectional area more than forced expiration in patients with tracheomalacia but not in healthy control subjects [10].

The tracheomalacia in this patient was too extensive for stenting. Evaluation was planned for nocturnal CPAP to protect against obstruction and hypoxemia during sleep. However, urgent BOTOX injection for symptomatic relief of achalasia was indicated and the plan was to proceed after prolonged NPO status.

Concerns in the anesthetic planning included airway protection from possible regurgitation of residual esophageal contents and the anticipation of possible dynamic tracheal collapse. There is no ideal approach. Some might consider rapid sequence induction (RSI) with cricoid pressure and intubation to be an optimal approach. The true risk of clinically significant aspiration in patients with achalasia is not known. Sellick's maneuver is not 100% effective in preventing aspiration and may in some patients actually displace the esophagus laterally or impair visualization of the glottic inlet [11]. A disadvantage of succinylcholine in the ambulatory surgery population is the possibility of profound myalgia. Moreover, in this patient, emergence and extubation are not straightforward. Incomplete evacuation of gastric contents and air following the procedure may potentiate the risk of regurgitation, particularly if deep extubation is attempted. Tracheal collapse distal to the endotracheal tube may also occur with cessation of positive pressure ventilation or with profound straining (Valsalva) in response to the endotracheal tube at emergence. A sedated fiberoptic intubation with the airway topicalized with local anesthetic could also be considered. This approach carries the disadvantage of a longer preparation time for planned repeat procedures and the risk of aspiration of gastric contents once the airway reflexes are ablated by local anesthetic and sedation that will carry over into the postextubation period.

Dexmedetomidine (DEX) is a selective, central *α*2-agonist with pharmacologic properties that include moderate sedation with anxiolysis, minimal respiratory depression, and preservation of airway reflexes. Compared with propofol, dexmedetomidine may better preserve upper airway tone [12]. The clinical effects of DEX are likely mediated through a central action in the locus coeruleus. Effective use of DEX for sedation in a variety of complex procedures, including fiberoptic intubation, has been demonstrated repeatedly in the literature, and the US FDA has recently expanded indications for DEX to include procedural sedation [13]. Ketamine is an NMDA receptor antagonist that provides amnesia and hypnosis through central dissociative mechanisms involving disruption of thalamic signaling pathways. Ketamine also promotes maintenance of spontaneous ventilation and preservation of airway reflexes. The combination of ketamine and racemic medetomidine is employed extensively for large animal surgery. A few recent literature reports describe the effective use of a ketamine-dexmedetomidine combination for procedural sedation [14–16]. 

We found the dexmedetomidine-ketamine combination to be highly effective in this circumstance. The patient was nonresponsive to passage of either scope yet maintained normal airway tone with no respiratory depression. The properties of dexmedetomidine do not lend it to use as a sole agent for invasive procedures that cause pain. Mild bradycardia and hypertension are associated with dexmedetomidine loading. Large, rapid bolus doses of dexmedetomidine (>2 mcg/kg) may be associated with effects on the ventilatory response to hypercarbia, airway tone, and hemodynamics. The combination of dexmedetomidine with additional agents for hypnosis, amnesia, or analgesia is optimal and may offset the limited side-effect profile of dexmedetomidine. Similarly, dexmedetomidine may balance the sialorrhea and sympathetic stimulation encountered with ketamine.

Fiberoptic visualization of the endoscopic procedure provided real-time airway assessment and a conduit for immediate airway rescue in the event of tracheal collapse. Vocal cord closure and cough reflex in response to saliva-based secretions in the glottic inlet were directly confirmed, thus demonstrating the maintenance of protective airway reflexes with this anesthetic regimen. Importantly, transnasal fiberoptic endoscopy demonstrated no dynamic upper airway collapse, during the procedure. However, in the event of intrathoracic tracheal collapse the flexible fiberoptic bronchoscope was positioned ready to advance through the collapsed segment after which oxygen could be delivered by jet ventilation via the suction port.

In summary, the management of patients with achalasia for endoscopic procedures presents numerous clinical challenges that are exacerbated by uncommon comorbidities such as tracheomalacia. The utility of dexmedetomidine in the management of this patient with a precarious airway and further evidence of the unique properties of a dexmedetomidine-ketamine combination in preservation of airway reflexes are discussed. This case offers an unconventional but efficacious and clinically sound approach to endoscopy in the setting of achalasia.
